# A group-based mental health intervention for Tanzanian youth living with HIV: Secondary analysis of a pilot trial

**DOI:** 10.1097/MD.0000000000028693

**Published:** 2022-02-18

**Authors:** Kalei R.J. Hosaka, Blandina T. Mmbaga, Aisa M. Shayo, John A. Gallis, Elizabeth L. Turner, Karen E. O’Donnell, Coleen K. Cunningham, Judith Boshe, Dorothy E. Dow

**Affiliations:** a John A. Burns School of Medicine, University of Hawaii at Manoa, Honolulu, HI; b Kilimanjaro Christian Medical Centre, Moshi, Tanzania; c Kilimanjaro Clinical Research Institute, Moshi, Tanzania; d Kilimanjaro Christian Medical University College, Moshi Tanzania; e Duke Global Health Institute, Duke University Medical Center, Durham, NC; f Department of Biostatistics & Bioinformatics, Duke University, Durham, NC; g Center for Health Policy and Inequalities Research, Duke University, Durham, NC; h Center for Child and Family Health, Durham, NC; i University of California, Irvine, Department of Pediatrics, Irvine, CA; j Division of Infectious Diseases, Department of Pediatrics, Duke University Medical Center, Durham, NC.

**Keywords:** mental health intervention, mental wellbeing, Tanzania, virologic suppression, youth

## Abstract

**Background:**

Youth living with human immunodeficiency virus (YLWH) are vulnerable to incomplete adherence to antiretroviral therapy in the context of stigma, decreased hope for future, and mental health challenges. Despite these challenges, few mental health interventions have been developed to support YLWH. Previous randomized results from the *Sauti ya Vijana* (SYV; “The Voice of Youth”) mental health intervention were indicative of the intervention's benefits in promoting virologic suppression.

**Methods:**

SYV is a group-based mental health and life skills intervention (pilot, individually randomized group treatment trial) developed alongside YLWH. SYV was comprised of 10, 90-minute sessions based on evidence-based treatment models designed to improve coping, social support, and hope for future as a pathway to improved adherence and virologic suppression. At baseline, YLWH 12 to 24 years of age were randomized to SYV or standard of care. Participants included in this secondary analysis were enrolled in SYV's crossover waves due to either being randomized to standard of care or inability to attend an earlier group, and therefore delayed intervention exposure. Measured outcomes included self-reported mental health measures, self-reported human immunodeficiency virus measures (stigma and adherence), and human immunodeficiency virus ribonucleic acid. Data was collected at baseline, preintervention, and postintervention timepoints. Participants were included if they attended a crossover wave and had data at all 3 timepoints.

**Results:**

Twenty-one crossover participants met inclusion criteria. Mean scores of self-reported mental health questionnaires were in an asymptomatic range both pre- and postintervention. Viral suppression was N = 15 (71%) preintervention compared to N = 17 (81%) postintervention. The participants who became virologically suppressed had no change in antiretroviral therapy.

**Conclusions:**

Despite the small sample size, findings from this study demonstrate that mental wellbeing may be an important pathway to improved HIV outcomes for YLWH. The same trend toward virologic suppression pre- to postintervention was demonstrated in the randomized pilot trial and suggests that SYV holds promise to improve HIV outcomes. Data from this analysis support the fully powered trial that is now underway.

## 1 Introduction

It is estimated that 4 million youth ages 15 to 24 years live with human immunodeficiency virus (HIV) (YLWH) globally, the vast majority of whom live in resource-limited settings.^[1]^ Approximately 190,000 YLWH live in Tanzania.^[2]^ Tanzania is home to 6% of the global population of adolescents who live with human HIV.^[3]^ Despite widespread availability of antiretroviral therapy (ART), 46,000 adolescents (ages 10–19 years) died due to AIDS-related complications in 2020 alone.^[4]^ Reducing mortality among YLWH will require continued improvement of testing services and retaining YLWH in care and treatment.^[4]^

Mental health support is critical in retaining YLWH in care and supporting their wellbeing.^[5]^ YLWH are at high risk for experiencing mental health problems.^[^6^,^^7^^]^ Mental health problems are associated with decreased adherence to ART and mortality for people living with HIV.^[5]^ Stigma, experiences of trauma, poverty, decreased caregiver support, and decreased hope for the future have also been linked with poor HIV outcomes.^[^8^,^^9^^]^ Nevertheless, while mental health is an important driver of HIV outcomes for YLWH, mental health services are limited and inaccessible for many – particularly in resource-limited settings.^[7]^ Moreover, few evidence-based mental health interventions exist to improve HIV outcomes for YLWH.

*Sauti ya Vijana* (SYV; “The Voice of Youth”) is a group-based mental health and life skills intervention developed in response to the complex psychosocial and mental health needs expressed during formative research with Tanzanian youth.^[^8^,^^10^^,^^11^^]^ Previous randomized results comparing intervention vs standard of care (SOC) arms were indicative of the intervention's benefits in promoting virologic suppression.^[11]^ In this report, we describe results from a secondary analysis of the pilot trial, comparing HIV and mental health outcomes pre- to postintervention for SYV's crossover wave participants (delayed exposure).

## 2 Methods

### 2.1 Study design

**Figure 1:**
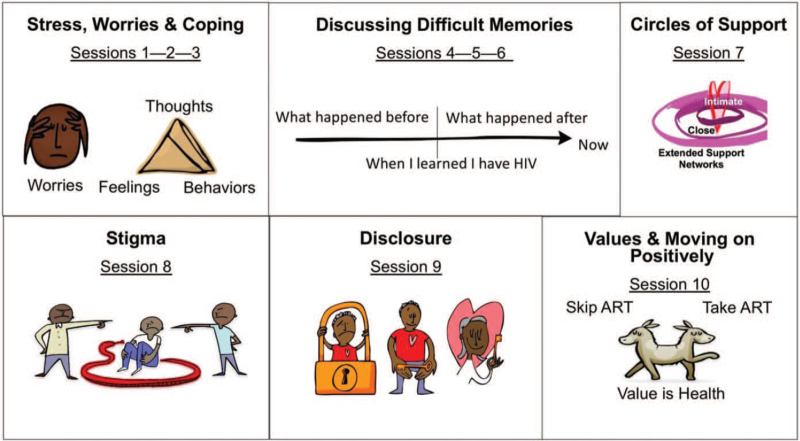
Sauti ya Vijana (SYV) intervention structure.

The SYV intervention was structured to include 10, 90-minute group-based sessions (2 with caregivers) and 2 individual sessions with group leaders (see Fig. 1). SYV sessions are based on existing evidence-based treatment models – including components of cognitive behavioral therapy, interpersonal psychotherapy, and motivational interviewing^[12]^ – designed to improve coping, resilience, social support, and hope for the future as a pathway to improved adherence and virologic suppression.^[11]^

This study was a pilot, individually randomized group treatment trial; this study used a stepped-wedge design that included 3 randomization waves and 3 crossover waves (see Figure, Supplemental Digital Content 1 which shows the trial's stepped-wedge design). This study was conducted at 2 adolescent clinic sites in Moshi, Tanzania: Kilimanjaro Christian Medical Centre and Mawenzi Regional Referral Hospital. Adolescents and young adults ages 12 to 24 years were recruited from these 2 adolescent clinics between May and July 2016. Study eligibility required participants receive ART and be aware of their HIV status. The pilot trial aimed to enroll approximately 100 participants in order to estimate intervention effectiveness and assess the intervention's feasibility and acceptability. Due to study attrition, enrollment was increased to 130 participants to meet the targeted sample size. The intervention was delivered by 6 young adults ages 23 to 30 years affected by and/or living with HIV.

### 2.2 Randomization and masking

While participants included in this analysis were part of the larger SYV randomized trial, this report focuses on pre- to postintervention change for participants who received the SYV intervention in 1 of 3 crossover waves (delayed exposure); this report does not report randomized results (for a detailed explanation of the randomization and masking process, see Dow et al^[11]^ publication in *BMC Public Health*). Briefly, at baseline, participants were organized by clinic site into 1 of 3 waves; each wave was comprised of 18 to 20 females and 18 to 20 males of similar age. Just prior to the start of each intervention wave, participants were randomized to SYV or SOC in the first 3 waves by coin flip. The SOC group did not meet together as part of the study but continued usual attendance to monthly clinics with peer youth engagement and adherence counselling.

### 2.3 Crossover intervention

**Figure 2:**
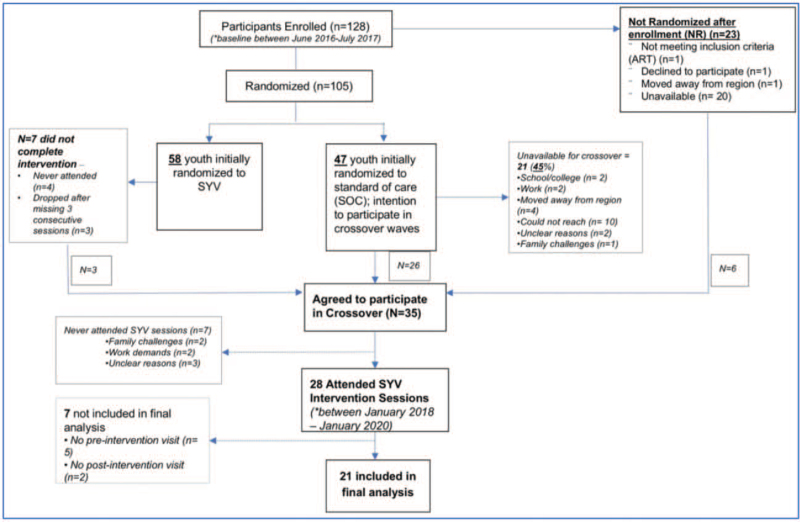
Flow diagram for SYV pilot trial, indicating crossover participation. Of 225 participants were assessed for eligibility, 128 enrolled. ^∗^Median difference between baseline and preintervention crossover visit for the 21 included in the final analysis was 1.8 years (interquartile range = 0.9–2.8 years). SYV = *Sauti ya Vijana* (“The Voice of Youth”) intervention.

After the intervention period for participants in SYV's first 3 waves, participants randomized to SOC were invited to receive the SYV intervention in 1 of 3 crossover waves as part of the stepped-wedge design (see Table, Supplemental Digital Content 2). Additionally, participants who initially enrolled but were unavailable for randomization (most of whom were in boarding school or working) or participants who randomized to SYV but had incomplete attendance (ie, never attended or prematurely discontinued SYV) were also invited to join a crossover wave (see Fig. 2, Flow Diagram, for the SYV pilot trial indicating crossover participation).

### 2.4 Measures and analysis

Quantitative measures were used to assess HIV and mental health outcomes pre- to postintervention. Outcomes included human immunodeficiency virus ribonucleic acid, self-reported mental health measures (patient health questionnaire-9, strengths and difficulties questionnaire, and University of California Los Angeles (UCLA) post-traumatic stress disorder reaction index), and self-reported HIV measures (stigma and adherence).^[^13^–^17^]^ Stigma was measured via a 10-question truncated Berger scale.^[14]^ Adherence was measured using a validated 3-question survey.^[13]^ Measures were administered in Kiswahili. Research assistants conducting study visits were blinded to intervention assignment, and no changes to methods were made during the course of the study. Timepoints included in this analysis were baseline (measurement at enrollment), preintervention, and postintervention (see Figure, Supplemental Digital Content 1 which shows the trial's stepped-wedge design). Due to our interest in average change from pre- to postintervention, participants were included if they attended 1 of 3 crossover waves and had data from these timepoints.

### 2.5 Statistics

We summarized continuous variables with means and standard deviations (SD); we summarized categorical variables with counts and percentages. Missing data as a result of nonresponse was less than 3%. If an item was skipped (eg, Strengths and Difficulties Questionnaire), the entire measure was considered missing. We also computed measure means and SDs with missing items imputed using the mean of other items in the measure, but this did not appreciably change the results. All statistical analyses were performed using Stata 16.1 (StataCorp LLC, College Station, TX).

### 2.6 Ethics

Participants provided written informed consent in Kiswahili; participants under 18 years provided assent while caregivers provided written consent. Participants were de-identified and assigned a study number. This study was approved by Duke University's Institutional Review Board, KCMC Ethics Committee, and the Tanzanian National Institute for Medical Research.

## 3 Results

The majority of participants who agreed to participate in the SYV crossover intervention came from the SOC group (N = 26, 74.3%). Six participants (17.1%) came from the group who initially enrolled in SYV but were unavailable for randomization, while 3 participants (8.6%) were initially randomized to SYV but did not complete the intervention. Of the 35 participants who agreed to participate in the crossover intervention, 21 participants met inclusion criteria of crossover attendance and data at all 3 timepoints (reference Fig. 2 for more detail). Those excluded had similar characteristics to those included in the analysis; however, a higher proportion of participants excluded were female (N = 9, 64.2%) (see Table, Supplemental Digital Content 2 which compares baseline characteristics of all participants who agreed to participate in crossover vs those included in the final analysis).

The median time from baseline to preintervention for participants was 1.8 years (interquartile range = 0.9–2.8). Median time from preintervention to postintervention was 0.4 year (interquartile range = 0.3–0.5). The mean age of participants at the preintervention timepoint was 20.5 years with a SD of 2.7 years. Thirteen (62%) of the 21 included participants were male and 19 (90%) were perinatally-infected.

The mean scores of all 3 self-reported mental health questionnaires (patient health questionnaire-9, strengths and difficulties questionnaire, and UCLA post-traumatic stress disorder reaction index) fell into an asymptomatic range both pre- and postintervention (Table 1). Despite 15% meeting criteria for moderate depressive symptoms at baseline based on a patient health questionnaire-9 score of 10 or greater, none met criteria immediately before nor after the intervention. Stigma and self-report adherence scores remained fairly consistent across all timepoints.

**Table 1:** HIV and mental health outcomes for 21 participants who agreed to participate in a crossover wave, attended the SYV intervention, and had data from all 3 timepoints^∗^.

	Baseline	Preintervention	Postintervention
Age	18.5 (2.4)	20.5 (2.7)	20.9 (2.7)
PHQ-9			
*Total score*	5.7 (3.8)	4.1 (2.7)	4.6 (3.0)
*≥10* ^†^	3 (15.0%)	0 (0%)	0 (0%)
SDQ			
*Total score*	7.9 (3.6)	6.9 (4.3)	6.7 (4.7)
*≥17* ^†^	0 (0%)	1 (4.8%)	1 (4.8%)
UCLA post-traumatic stress disorder reaction index			
*Total score*	9.0 (6.8)	7.1 (5.3)	8.8 (7.3)
*≥18* ^†^	3 (14.3%)	0 (100%)	2 (9.5%)
Stigma			
*Total score*	22.6 (2.9)	21.8 (4.5)	21.9 (4.3)
*Internal score*	7.8 (1.4)	7.4 (1.4)	7.3 (1.4)
*External score*	15.2 (3.6)	14.3 (4.2)	14.5 (4.0)
Adherence (self-report score)	61.4 (11.2)	64.4 (12.0)	61.8 (12.3)
Viral load copies/mL			
*Total score Log*_*10*_	5.1 (2.9)	5.1 (3.3)	4.3 (2.6)
*Virologic suppression* ^†^ *(ie, HIV–RNA <* *400 copies/mL)*	13 (61.9%)	15 (71.4%)^‡^	17 (81.0%)

HIV-RNA = human immunodeficiency virus ribonucleic acid, PHQ-9 = patient health questionnaire-9, SDQ = strengths and difficulties questionnaire, SYV = *Sauti ya Vijana* (“The Voice of Youth”) intervention.

∗ Means (standard deviations) and n (%) are reported unless otherwise noted.

† Reported as count (percentage).

‡ One participant's preintervention study viral load could not be quantified due to an inhibitor in the sample. This participant was analyzed as being virologically suppressed due to having an undetectable viral load at all study visits and within 6 mo as part of standard of care. Missing Single Item questionnaire response from baseline: 1 participant for PHQ-9; 4 participants for SDQ; 2 for stigma and internal stigma.

Fifteen participants (71%) were virologically suppressed (human immunodeficiency virus ribonucleic acid < 400 copies/mL) preintervention; 17 participants were virologically suppressed (N = 17, 81%) postintervention. Those participants with virologic failure preintervention who became virologically suppressed postintervention remained on the same protease-inhibitor based regimen (atazanavir/ritonavir or lopinavir/ritonavir). Two participants did change from atazanavir/ritonavir to dolutegravir preintervention to postintervention, but their virologic status did not change.

No harms or unintended effects of the intervention were found among participants in this analysis.

## 4 Discussion

Supporting the mental wellbeing of YLWH is critical to improving adherence to ART and HIV outcomes and decreasing risk of HIV transmission.^[8]^ Despite our small sample, we observed a trend toward virologic suppression pre- to postintervention without change in ART. This trend is consistent with the 10% virologic suppression gain previously demonstrated across randomized arms (intervention arm vs SOC).^[11]^ Dolutegravir – a potent integrase inhibitor – was introduced in Tanzania in 2019. Over the course of the study, several participants shifted to dolutegravir to increase potency and reduce pill burden; however, the participants who became virally suppressed following the intervention continued receiving the same protease-inhibitor-based regimen, suggesting suppression was a result of improved adherence.

Limitations of this study include low numbers of crossover participants with complete data to meet inclusion criteria and no control group of participants during the crossover waves. Crossover participants attended the same clinic as those initially randomized to the intervention arm. It is possible that “spill-over” of intervention content occurred during the preintervention period.

## 5 Conclusion

Despite caution with our small sample size, findings from this study reinforce that mental wellbeing may be an important pathway to improved HIV outcomes for YLWH. We observed a trend toward virologic suppression pre- to postintervention without change in ART. Together, these data suggest that SYV holds promise to improve HIV outcomes for YLWH and support the definitive trial that is now underway.

## Acknowledgments

The authors would like to thank the SYV group leaders for their compassion, effort, and dedication to the project; study participants for their commitment and resilience; and clinic staff who supported the study.

## Author contributions

**Conceptualization:** Blandina T. Mmbaga, Aisa M. Shayo, Karen E. O’Donnell, Coleen K. Cunningham, Dorothy E. Dow.

**Data curation:** Kalei Richard James Hosaka, John A. Gallis, Elizabeth L. Turner, Dorothy E. Dow.

**Formal analysis:** Kalei Richard James Hosaka, John A. Gallis, Elizabeth L. Turner.

**Funding acquisition:** Dorothy E. Dow.

**Investigation:** Dorothy E. Dow.

**Methodology:** Dorothy E. Dow.

**Project administration:** Dorothy E. Dow.

**Supervision:** Blandina T. Mmbaga, Judith Boshe, Dorothy E. Dow.

**Writing – original draft:** Kalei Richard James Hosaka.

**Writing – review & editing:** Kalei Richard James Hosaka, Blandina T. Mmbaga, Aisa M. Shayo, John A. Gallis, Elizabeth L. Turner, Karen E. O’Donnell, Coleen K. Cunningham, Judith Boshe, Dorothy E. Dow.

## Supplementary Material

Supplemental Digital Content

## Supplementary Material

Supplemental Digital Content
