# Supplementary material for: A group-based mental health intervention for Tanzanian youth living with HIV: Secondary analysis of a pilot trial
Source: Medicine (Baltimore). 2022 Feb 18;101(7):e28693. doi: 10.1097/MD.0000000000028693 (PMC9282032; doi:10.1097/MD.0000000000028693)
Supplement: Supplemental Digital Content [file medi-101-e28693-s001.docx]

**
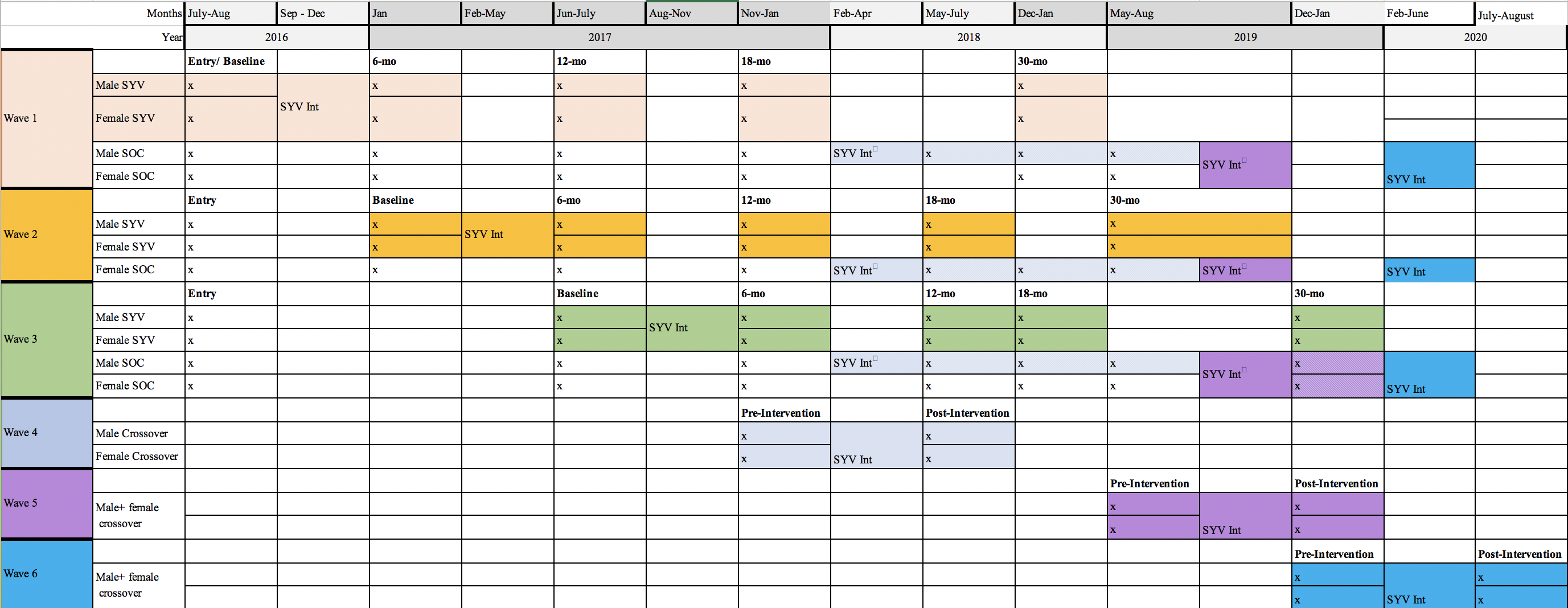
**

**Supplemental Digital Content 1 (Figure): Sauti ya Vijana Pilot Trial (2016-2020), Stepped-Wedge Study Design**

Baseline visits for crossover wave participants occurred between June 2016 and July 2017 (i.e., baseline for wave 1, wave 2, or wave 3). Pre-intervention visits varied by crossover wave and occurred between Nov. 2017 and January 2020. Median from baseline to pre-intervention for participants was 1.8 years (Interquartile Range=0.9 to 2.8). Median time from pre-intervention to post-intervention was 0.4 year (Interquartile Range=0.3 to 0.5) (range: May 2018 to Aug 2020).

Abbreviations: SYV= Sauti ya Vijana intervention; SOC= standard of care “control” group; mo=month. Baseline includes 88 participants; an additional 40 participants enrolled over time to complete groups for waves 2 and 3.

Shading: Wave 1 in pink, Wave 2 in orange (note, no male SOC group), Wave 3 in green, Wave 4 in blue, Wave 5 in purple, Wave 6 in turquoise with study follow visits shaded for those who received intervention and remaining clear for the control group; not all crossed over. First crossover wave in blue; second crossover wave in purple (run as a mixed gender and mixed site group); third crossover wave in turquoise.
