# Supplementary material for: A group-based mental health intervention for Tanzanian youth living with HIV: Secondary analysis of a pilot trial
Source: Medicine (Baltimore). 2022 Feb 18;101(7):e28693. doi: 10.1097/MD.0000000000028693 (PMC9282032; doi:10.1097/MD.0000000000028693)
Supplement: Supplemental Digital Content [file medi-101-e28693-s002.docx]

**Supplemental Digital Content 2 (Table)**. Baseline characteristics of all participants who agreed to participate in crossover vs. participants included in the final analysis^a^

|  | All Who Agreed to SYV Crossover (n=35) | Included in Final Analysis (n=21) |
| --- | --- | --- |
| Age (mean, SD) | 18.6 (2.3) | 18.5 (2.4) |
| Sex, female^b^ | 17 (48.6%) | 8 (38.1%) |
| Perinatally-Infected^b^ | 33 (94.3%) | 19 (90.5%) |
| Age at diagnosis^c^ | 8.6 (3.2) | 8.2 (3.5) |
| Socioeconomic Status^b^ |  |  |
| House Has Electricity | 27 (77.1%) | 18 (85.7) |
| House has indoor plumbing | 21 (60.0%) | 12 (57.1%) |
| Owns a Cell Phone | 25 (71.4%) | 14 (66.7%) |
| Primary Caregiver^b^ |  |  |
| Mom or Dad | 16 (45.7%) | 9 (42.9%) |
| Aunt or Uncle | 12 (34.3%) | 8 (38.1%) |
| Grandmother or Grandfather | 3 (8.6%) | 2 (9.5%) |
| Brother or Sister | 3 (8.6%) | 2 (9.5%) |
| Other, unspecified | 1 (2.9%) | 0 (0.0%) |
| Patient Health Questionnaire-9 (PHQ-9)^c^ | |  |
| *Total score* | 6.0 (3.9) | 5.7 (3.8) |
| *>10*^b^ | 5 (15.2%) | 3 (15.0%) |
| *Total score* | 7.3 (3.7) | 7.9 (3.6) |
| *>17*^b^ | 0 (0%) | 0 (0%) |
| UCLA Post-Traumatic Stress Disorder Reaction Index | |  |
| *Total score* | 9.6 (7.3) | 9.0 (6.8) |
| *>18*^b^ | 5 (14.3%) | 3 (14.3%) |
| Stigma |  |  |
| *Total score*^c^ | 22.7 (4.4) | 22.6 (2.9) |
| *Internal score* | 7.9 (1.8) | 7.8 (1.4) |
| *External score*^c^ | 15.1 (3.9) | 15.2 (3.6) |
| Adherence (self-report score) | 59.0 (14.9) | 61.4 (11.2) |
| Viral Load Copies/mL^d^ |  |  |
| *Total score Log_10_* | 4.9 (2.7) | 5.1 (2.9) |
| *Virologic suppression*^b^ *(i.e., HIV RNA < 400 copies/mL)* | 22 (66.7%) | 13 (61.9%) |

^a^Means (standard deviations) and n (%) are reported unless otherwise noted.

^b^Reported as count (percentage)

^c^Missing single item questionnaire response: for Column 1 (All Who Agreed to SYV Crossover), 1 participant for age at diagnosis; 2 participants for PHQ-9; 4 participants for SDQ-9; 2 participants for total stigma score and internal stigma. For Column 2 (All Who Attended SYV Crossover), 1 participant for age at diagnosis; 1 participant for PHQ-9; 4 participants for SDQ; 2 participants for total stigma score and internal stigma; 1 for viral load. For Column 3 (Included in Final Analysis), 1 participant for age at diagnosis; 1 participant for PHQ-9; 4 participants for SDQ; 2 for stigma and internal stigma.

^d^Missing virologic data at baseline: for Column 1, 2 participants for viral load. For Column 2, 1 participant for viral load.

List of Abbreviations: PHQ-9 (Patient Health Questionnaire-9), SDQ (Strengths and Difficulties Questionnaire).
